# Supplementary material for: Crystal structures and insights into precursor tRNA 5’-end processing by prokaryotic minimal protein-only RNase P
Source: Nat Commun. 2022 Apr 28;13:2290. doi: 10.1038/s41467-022-30072-6 (PMC9051087; doi:10.1038/s41467-022-30072-6)
Supplement: Supplementary file 1 — Supporting information [file 41467_2022_30072_MOESM1_ESM.pdf]

Supporting Information

For

Crystal structures and insights into precursor tRNA 5'-end processing by prokaryotic minimal protein-only RNase P

Yangyang Li[1], Shichen Su[2], Yanqing Gao[1], Guoliang Lu[2], Hehua Liu[1], Xi Chen[1], Zhiwei Shao[1], Yixi Zhang[1], Qiyuan Shao[1], Xin Zhao[1], Jie Yang[1], Chulei Cao[1], Jinzhong Lin[2], Jinbiao Ma[2], Jianhua Gan[1,*]

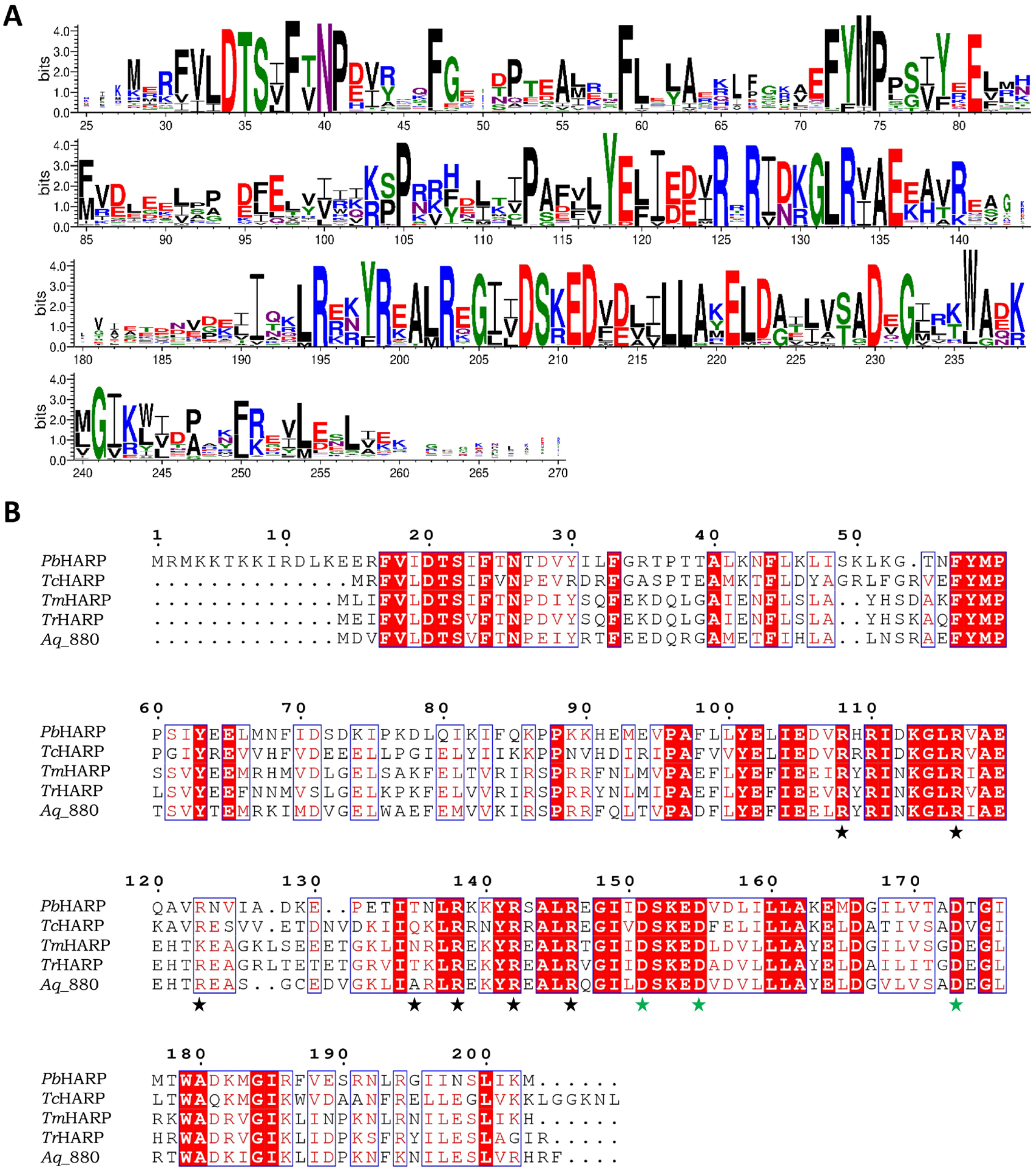

Supplementary Fig. 1: Sequence conservation and alignment of HARPs. A) WebLogo to illustrate amino acid conservation of HARPs. **B**) Sequence alignment of HARPs encoded by *lanctomycetes bacterium* GWF2_40_8 (*P. bacterium*), *Thermococcus celer* (*T. celer*), *Thermocrinis minervae* (*T. minervae*), *Thermocrinis ruber* (*T. ruber*), and *Aquifex aeolicus*.

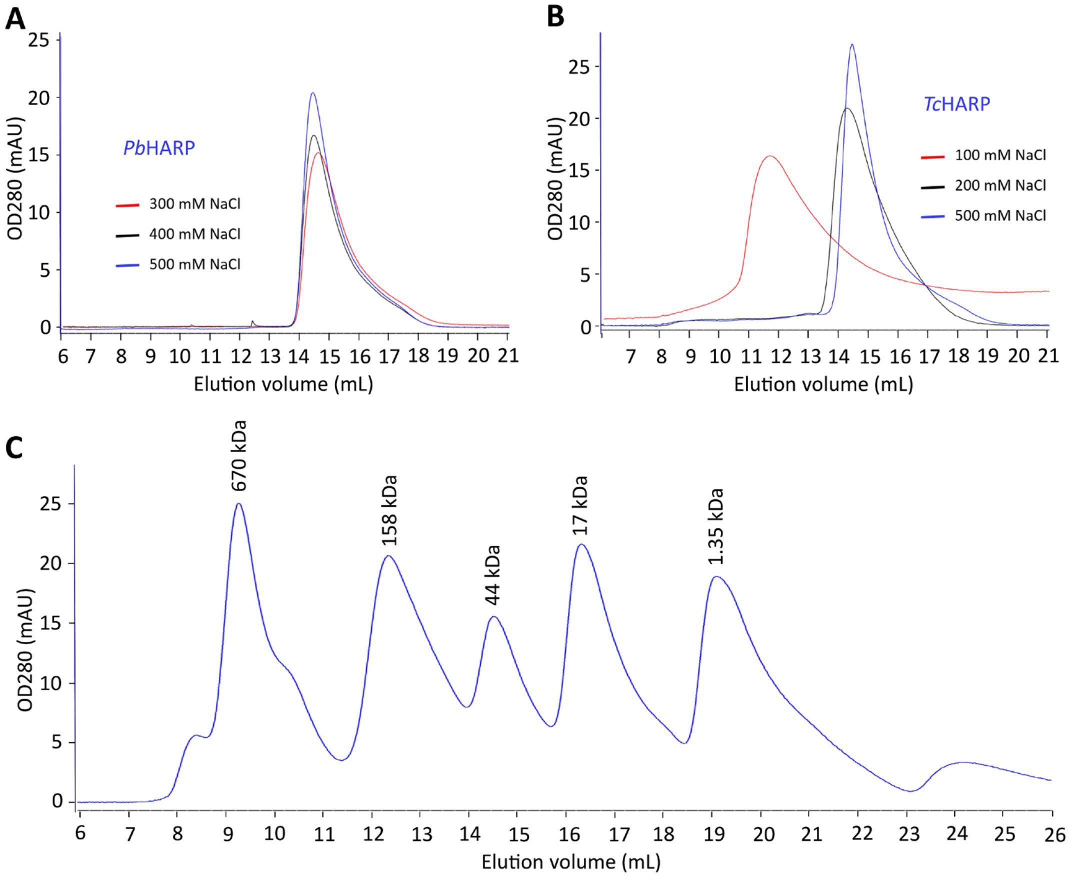

Supplementary Fig. 2: Size-exclusion chromatographic analysis of HARP proteins. **A**) Size-exclusion chromatographic analysis of *Pb*HARP. **B**) Size-exclusion chromatographic analysis of *Tc*HARP. **C**) Size-exclusion chromatographic analysis of standard marker proteins run on the same Superdex 200 Increase 10/300 GL column. For experimental details, see Methods section of the main text. Source data are provided in Source Data file.

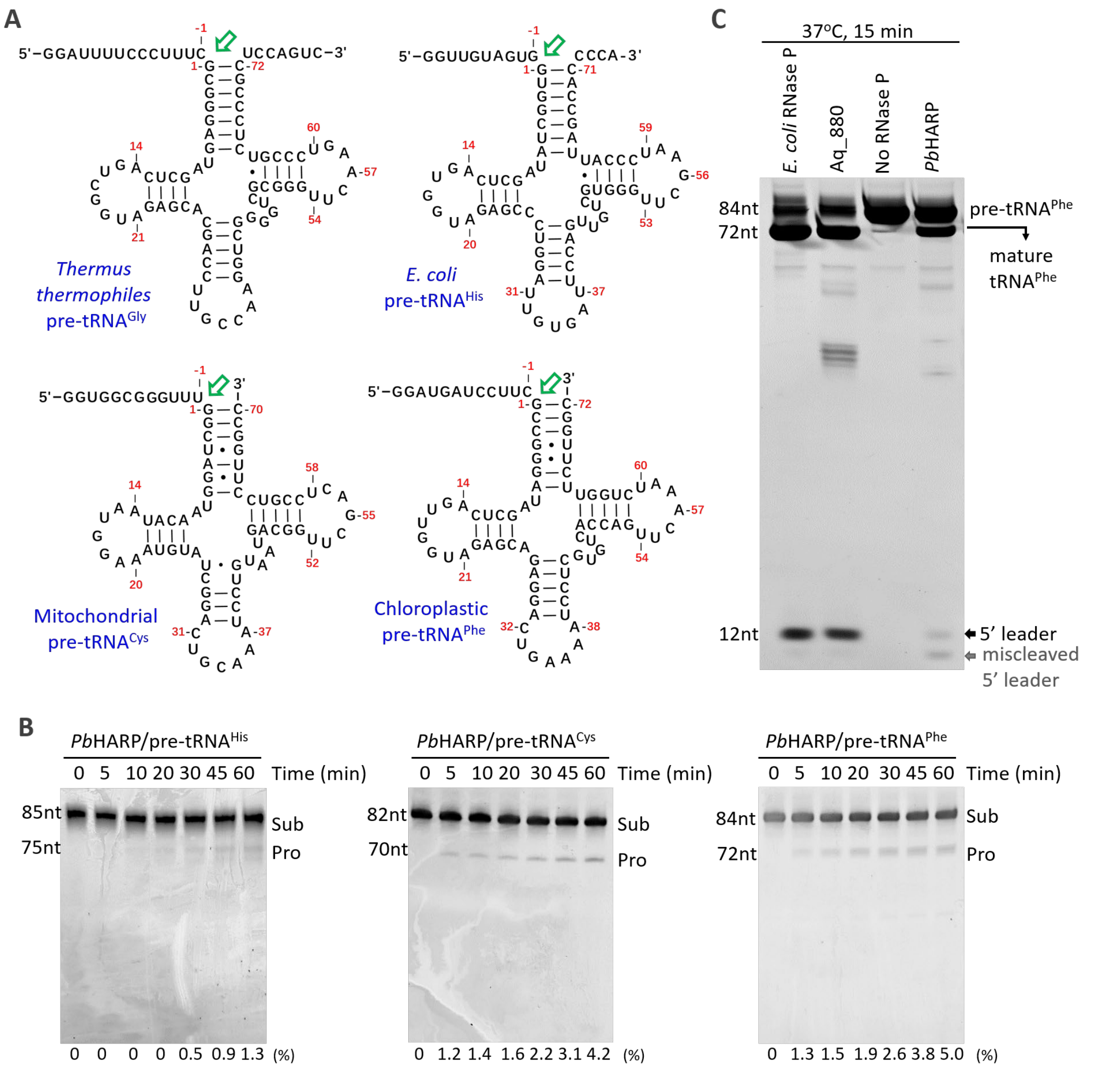

Supplementary Fig. 3: Sequence and *in vitro* cleavage assays of pre-tRNA. A) Sequences of pre-tRNA^{Gly}, pre-tRNA^{His}, pre-tRNA^{Cys} and pre-tRNA^{Phe}. **B)** *In vitro* pre-tRNA cleavage assays catalyzed by *Pb*HARP. The concentrations are 1 μM and 0.05 μM for pre-tRNA and *Pb*HARP, respectively. **C)** *In vitro* pre-tRNA^{Phe} cleavage assays catalyzed by *Pb*HARP, Aq_880 and *E. coli* RNase P. The concentrations are 5 μM and 0.25 μM for pre-tRNA and RNase P, respectively. The reaction mixtures are separated on 10% and 20% denaturing UREA-PAGE gels in panels **B** and **C**, respectively. The substrate cleavage percentage (%) is shown at the bottom of the gels. Experiments were repeated independently twice (**B** and **C**) with similar results. Source data are provided in Source Data file.

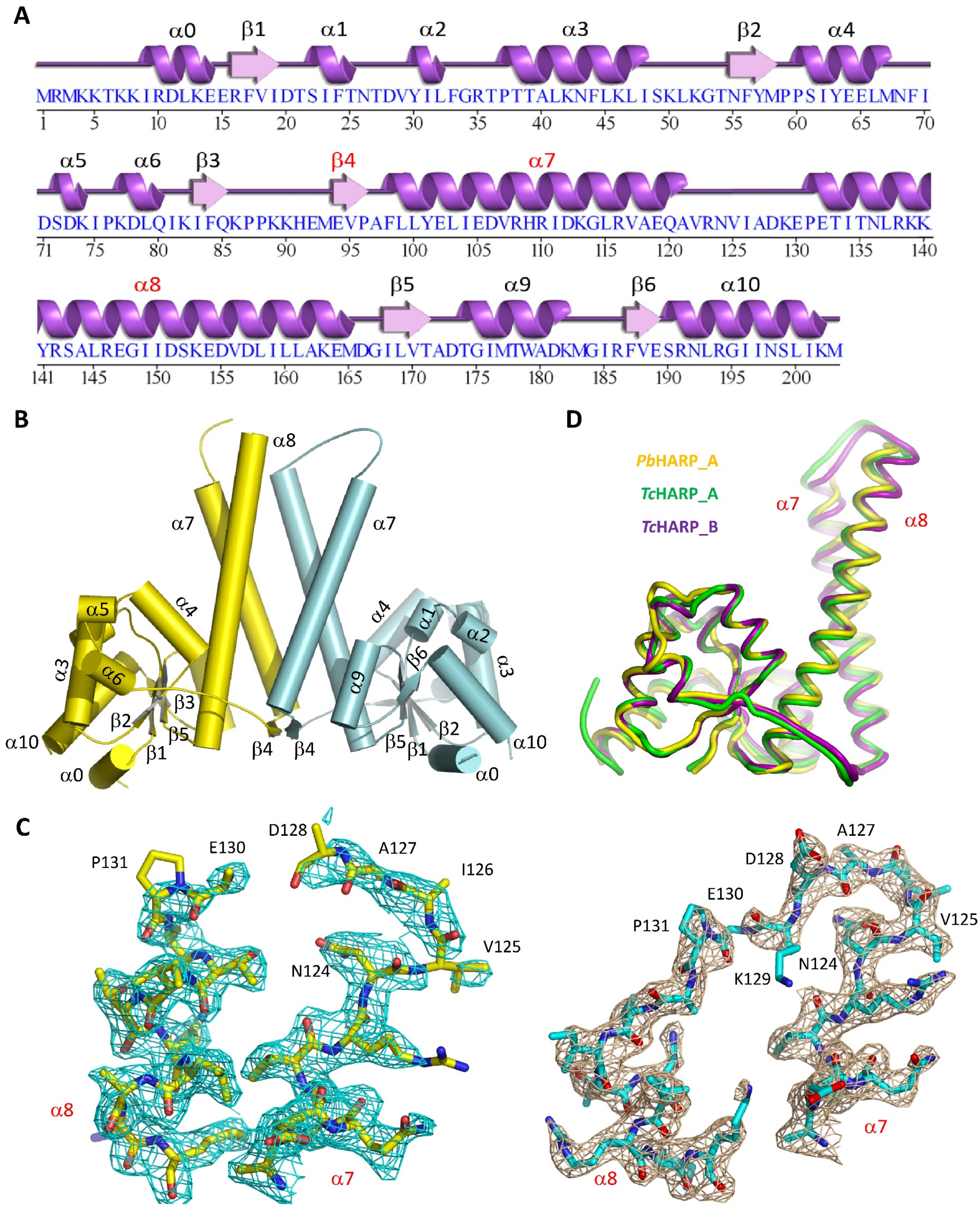

Supplementary Fig. 4: Apo-form Structure of HARPs. A) Sequence and secondary structure of apo-form *Pb*HARP. **B**) Cartoon presentation showing the overall fold of apo-form *Pb*HARP. **C)** The detailed conformations and $2F_o$-F_c electron density maps of the α7-α8 connecting linker in the apo-form *Pb*HARP structure. The maps are contoured at 1.0 σ level. **D**) Structural superposition of *Pb*HARP and *Tc*HARP monomers.

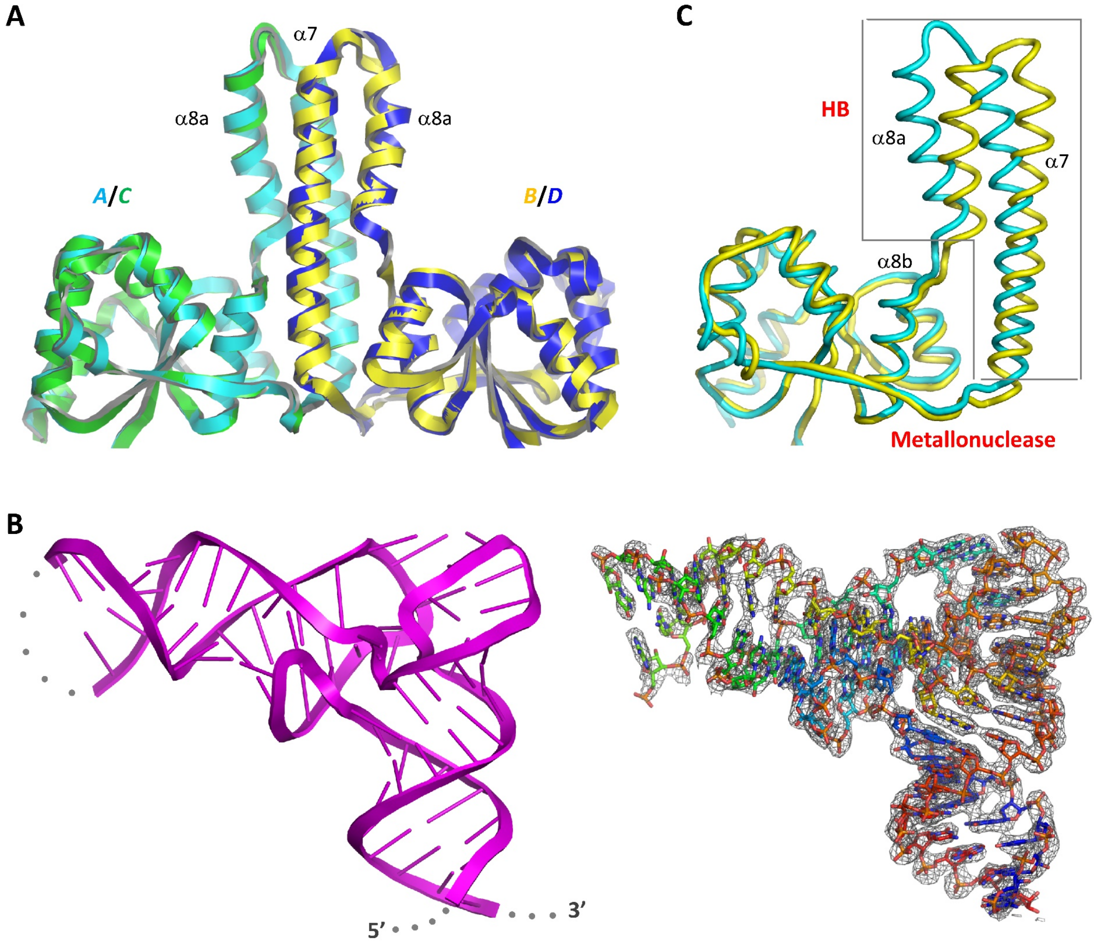

Supplementary Fig. 5: Complex structure of *Pb*HARP/pre-tRNA^{His}. A) Superposition of the AB and CD dimers of *Pb*HARP. The AB dimer is colored in cyan and yellow, whereas the CD dimer is colored in green and blue. **B**) Folding and electron density maps of pre-tRNA^{His}. The $2F_o$-F_c electron density maps are contoured at 1.0 σ level. **C**) Superposition of the A and B monomers of *Pb*HARP, which are colored in cyan and yellow, respectively.

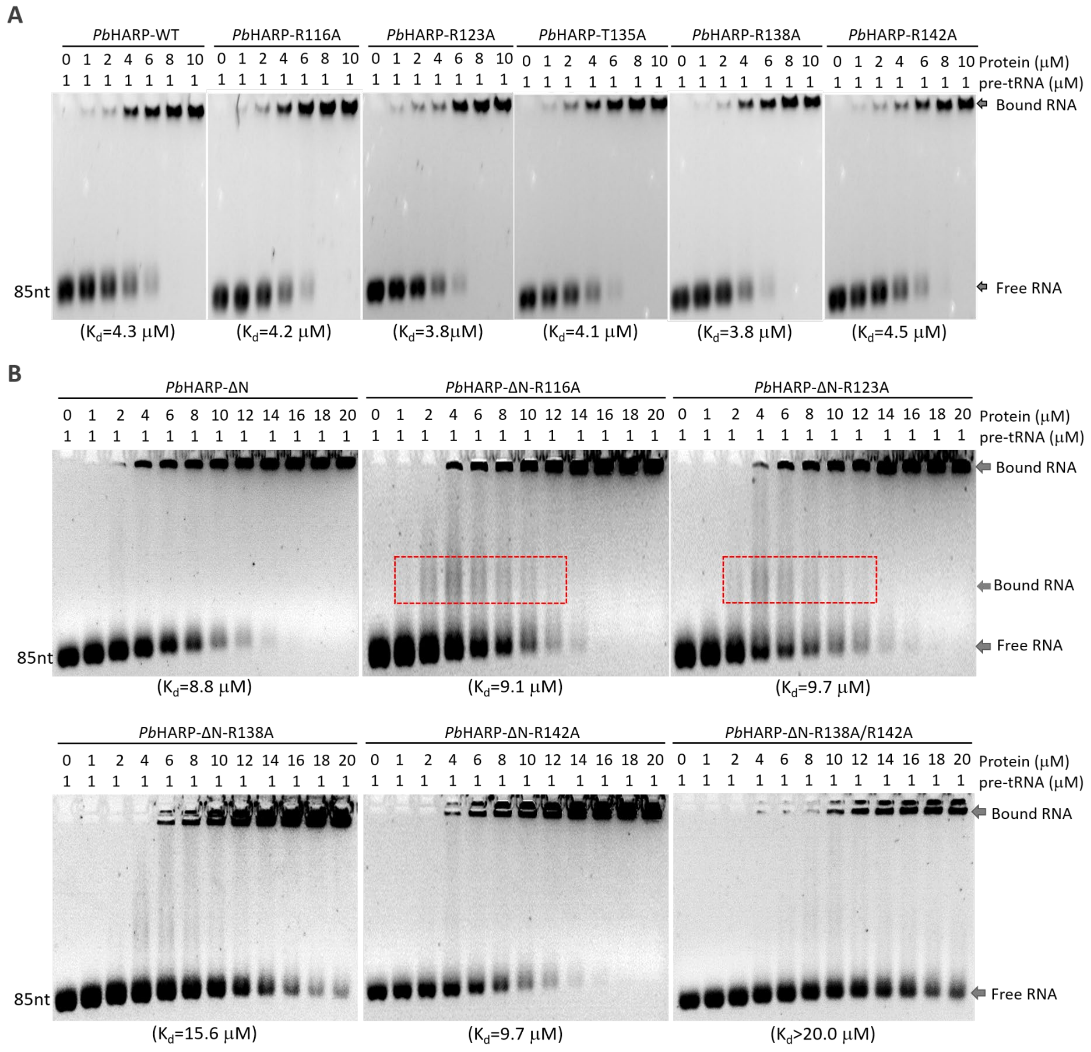

Supplementary Fig. 6: Comparison of *in vitro* pre-tRNA binding by WT and mutated *Pb*HARP proteins. A) Pre-tRNA^{His} binding by the full-length WT *Pb*HARP and mutants with mutation of the pre-tRNA recognizing residues. **B**) Pre-tRNA^{His} binding by WT and mutated *Pb*HARP proteins with N-terminal truncation. The estimated K_d values were listed at the bottom of the gels. Experiments were repeated independently three times with similar results. Source data are provided in Source Data file.

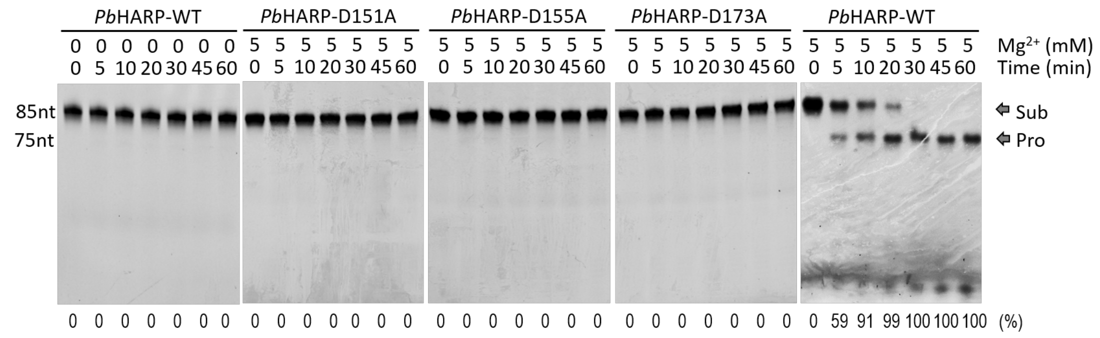

Supplementary Fig. 7: *In vitro* pre-tRNA^{His} cleavage assays showing the functional importance of divalent metal ions (Mg^{2+}) and the catalytic Asp residues of *Pb*HARP. The substrate cleavage percentage (%) is shown at the bottom of the gels. For experimental details, see Methods section of the main text. Experiments were repeated independently three times with similar results. Source data are provided in Source Data file.

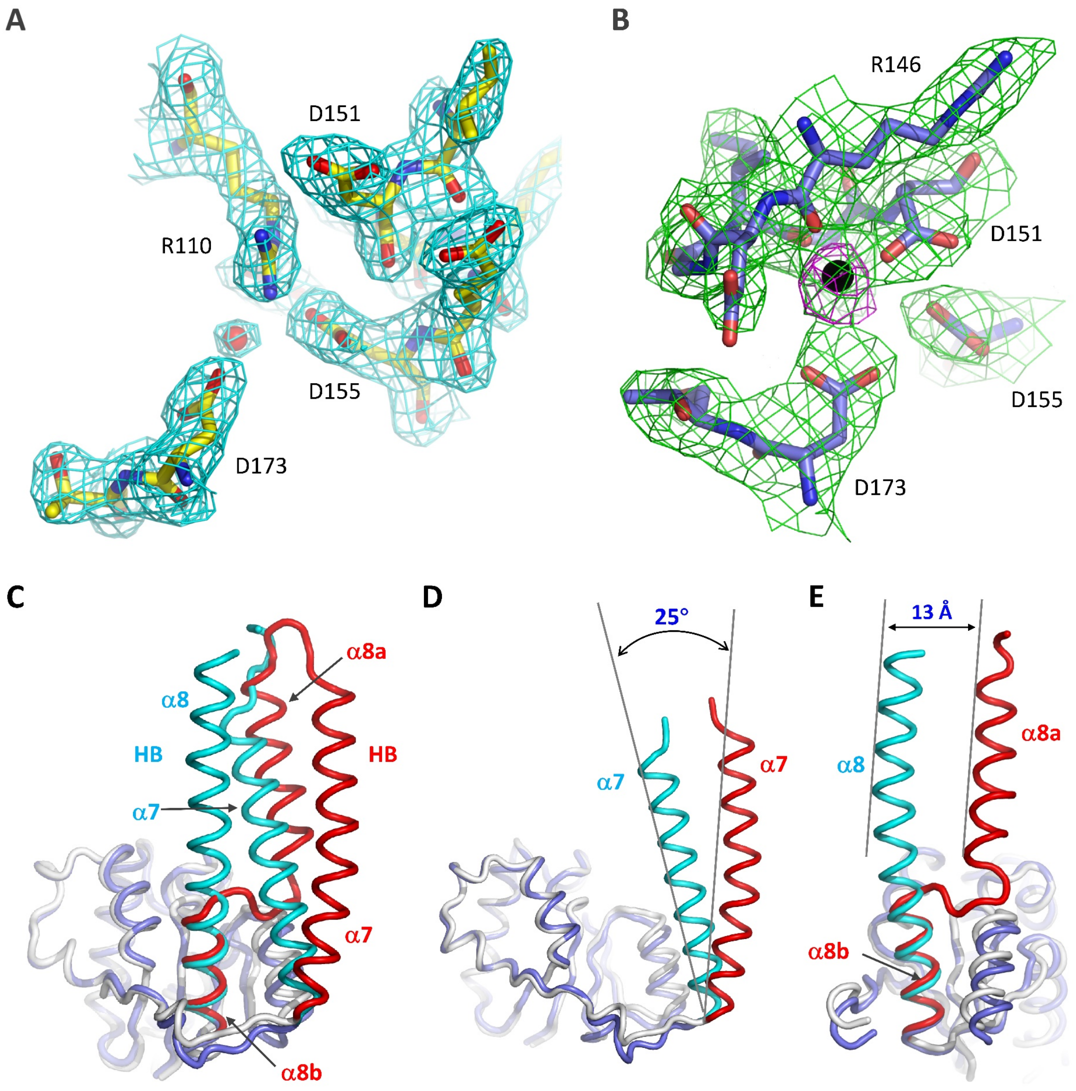

Supplementary Fig. 8: Conformational changes of *Pb*HARP. A) Conformation of the catalytic Asp residues in the apo-form structure. The $2F_o$-F_c electron density maps are contoured at 1.0 σ level. **B**) Conformation of the catalytic Asp residues in the pre-$tRNA^{His}$-complexed structure. The $2F_o$-F_c electron density maps are contoured at 1.0 σ level and colored in green. The F_o-F_c omit map of Ca^{2+} is contoured at 4.0 σ level and colored in magenta. Ca^{2+} is shown as black sphere**. C-E**) Superposition showing the conformational changes of *Pb*HARP HB domains. *Pb*HARP in the apo-form structure is colored in white and cyan, whereas it is colored in blue and red in the complex structure.

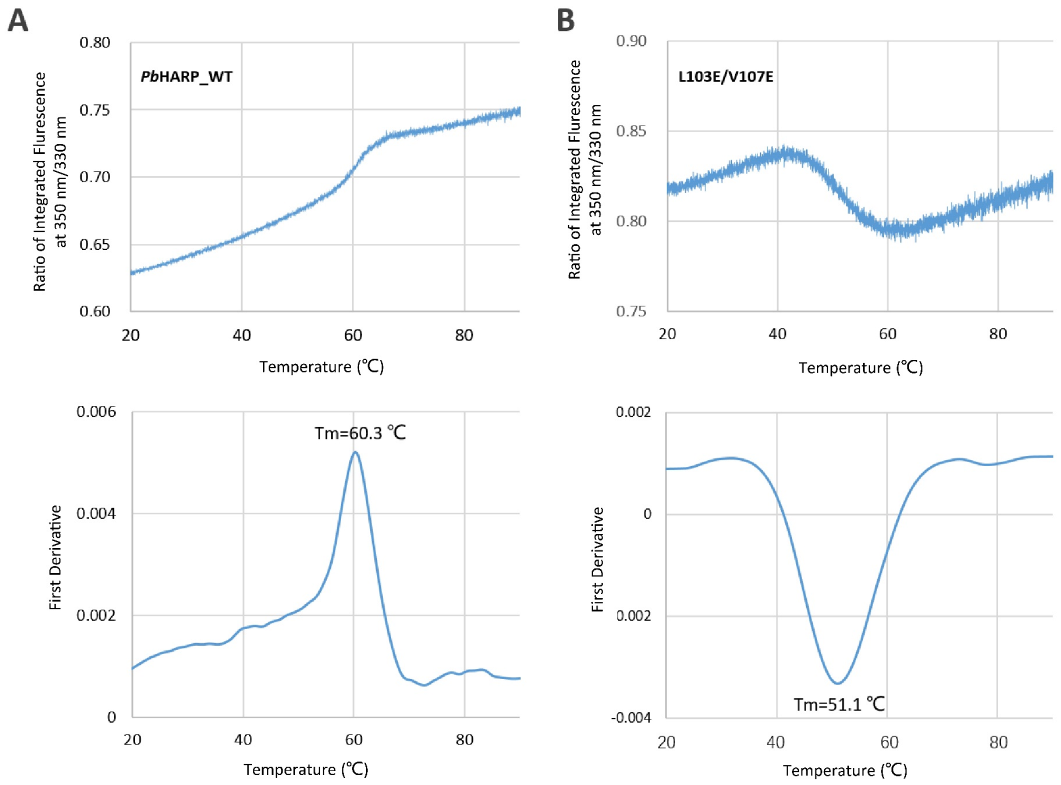

Supplementary Fig. 9: Nano-DSF analysis of *Pb*HARP proteins. A) Nano-DSF signal ratio 350 nm/330 nm and its first derivative plots of WT *Pb*HARP in function of the temperature. **B)** Nano-DSF signal ratio 350 nm/330 nm and its first derivative plots of the L103E/V107E mutant of *Pb*HARP in function of the temperature. Both proteins are dissolved in buffer composed of 20 mM Tris pH 8.0, 500 mM NaCl and 5% glycerol. The concentrations of proteins are 1.0 mg/mL. Source data are provided in Source Data file.

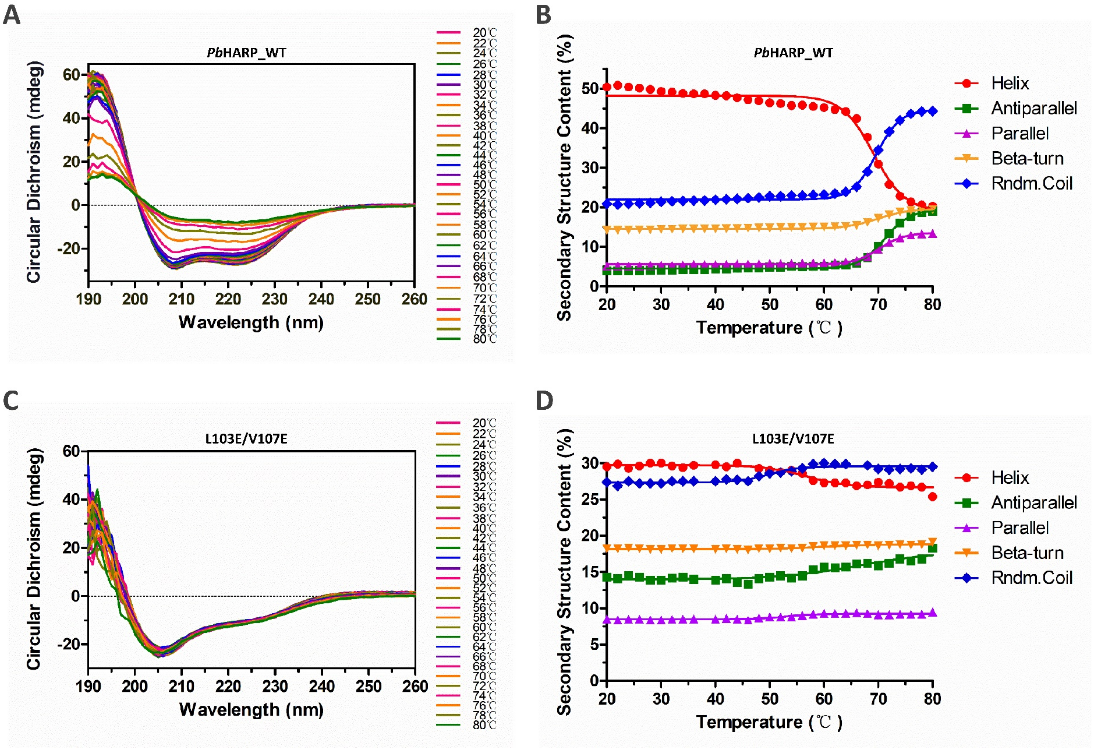

Supplementary Fig. 10: CD spectra analysis of *Pb*HARP proteins. A-B) CD spectra and secondary structure content of WT *Pb*HARP in function of the temperature. **C-D)** CD spectra and secondary structure content of the L103E/V107E mutant of *Pb*HARP in function of the temperature. Both proteins are dissolved in buffer composed of 5 mM Tris pH 8.0, 500 mM NaF. The concentrations of proteins are 0.2 mg/mL. Source data are provided in Source Data file.

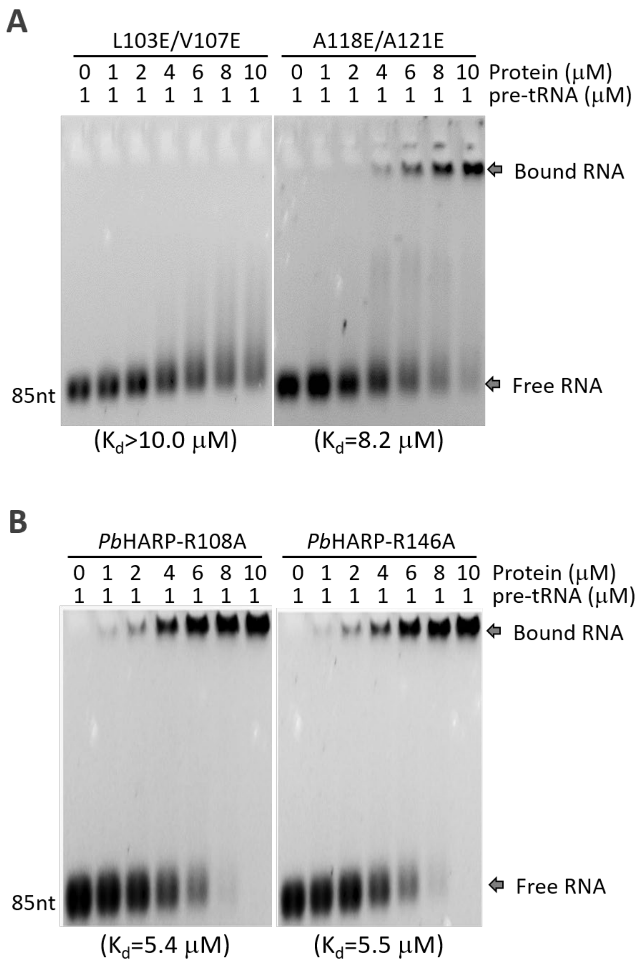

Supplementary Fig. 11: *In vitro* pre-tRNA binding by *Pb*HARP mutant proteins. A) Pre-tRNA^{His} binding by L103E/V107E and A118E/A121E mutants of *Pb*HARP. **B**) Pre-tRNA^{His} binding by R108A and R146A mutant proteins of *Pb*HARP. The estimated K_d values were listed at the bottom of the gels. Experiments were repeated independently three times with similar results. Source data are provided in Source Data file.

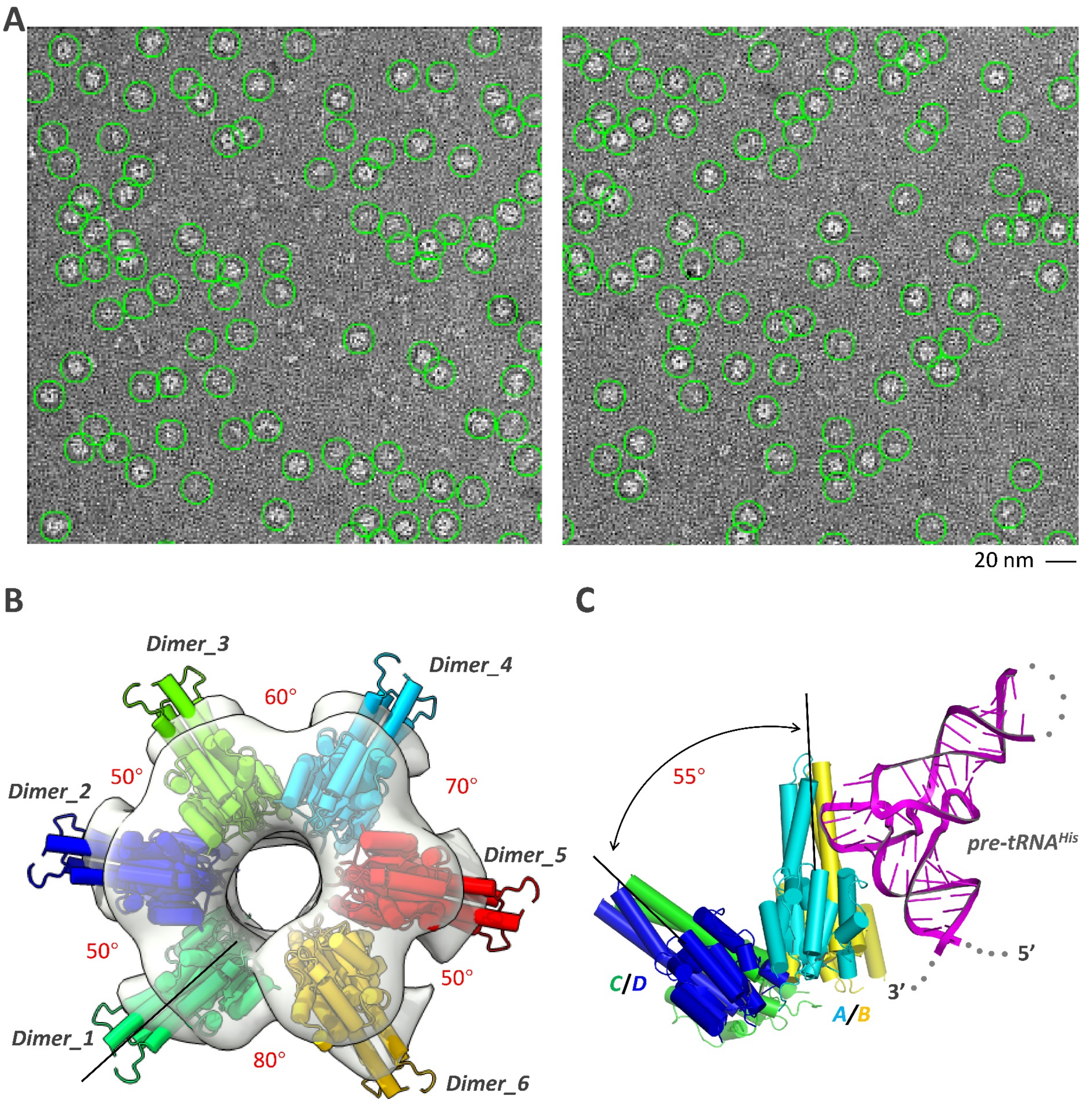

Supplementary Fig. 12: Assembly of HARP. A) Enlarged negative stain CCD image of *Tr*HARP. Experiments were repeated independently twice with similar results. **B**) The electron microscopy density maps and the modeled structure of *Tr*HARP. The rotation angle between neighboring *Tr*HARP dimers are shown in the figure. **C**) Cartoon presentation showing the assembly and rotation angle between the AB and CD *Pb*HARP dimers in the pre-tRNA^{His} complexed structure.

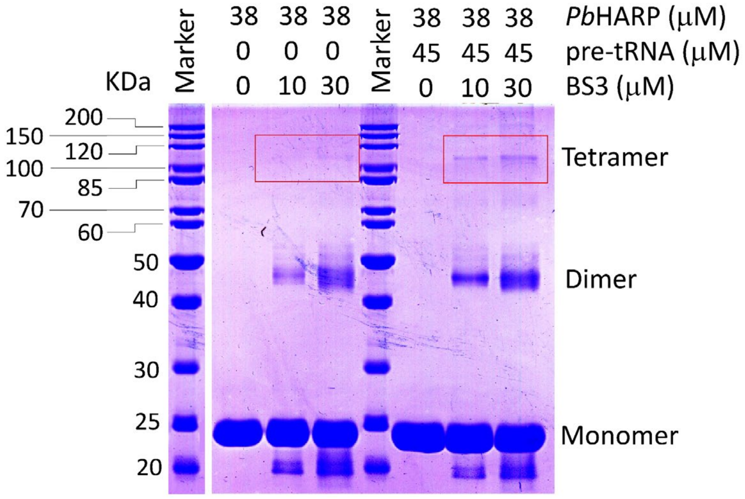

Supplementary Fig. 13: The BS3 crosslinking of *Pb*HARP. The His-7bp pre-tRNA concentrations are 45 μM if present. The concentrations of BS3 and *Pb*HARP are indicated on the figure. Experiments were repeated independently twice with similar results. Source data are provided in Source Data file.

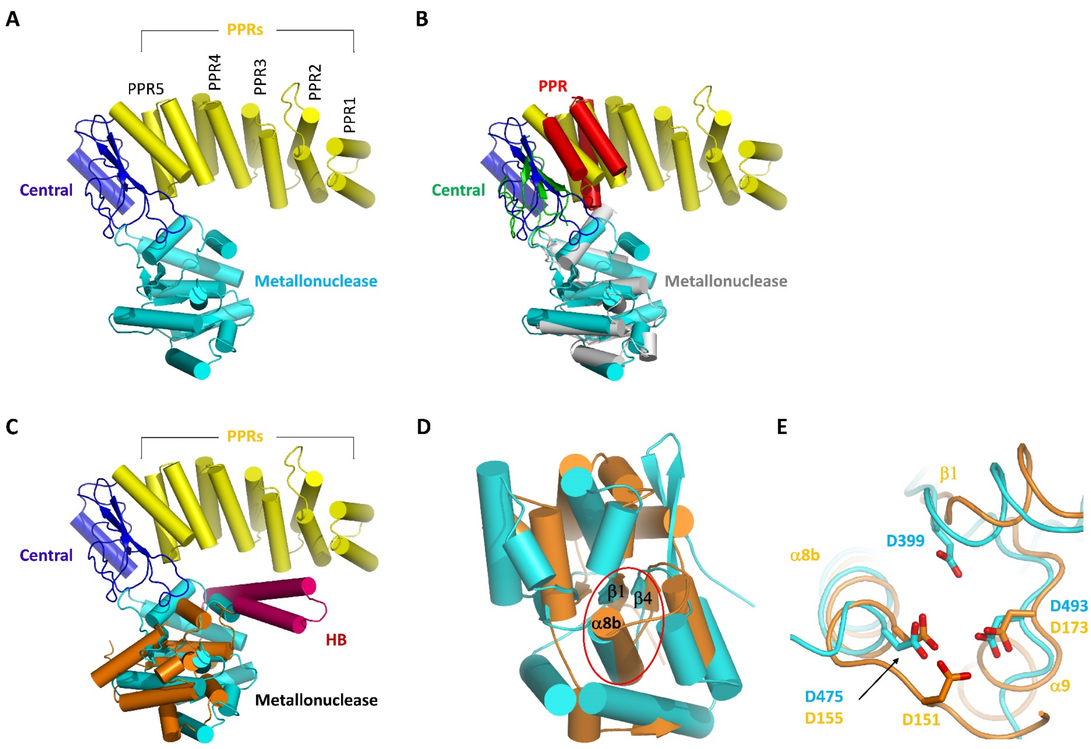

Supplementary Fig. 14: Comparison of different classes of PRORPs. A) The overall structure of apo-form *At*PRORP1. **B**) Structural superposition of *At*PRORP1 and *Hs*MRPP3, which is colored in white, green, and red. **C**) Structural superposition of *At*PRORP1 and the pre-tRNA^{His}-complexed *Pb*HARP, which is colored in brown and magenta. **D**) Superposition of the metallonuclease domains of *At*PRORP1 and *Pb*HARP. **E**) Comparison of the catalytic Asp residues of *At*PRORP1 and *Pb*HARP. *At*PRORP1 is colored in cyan, blue, and yellow in all panels.

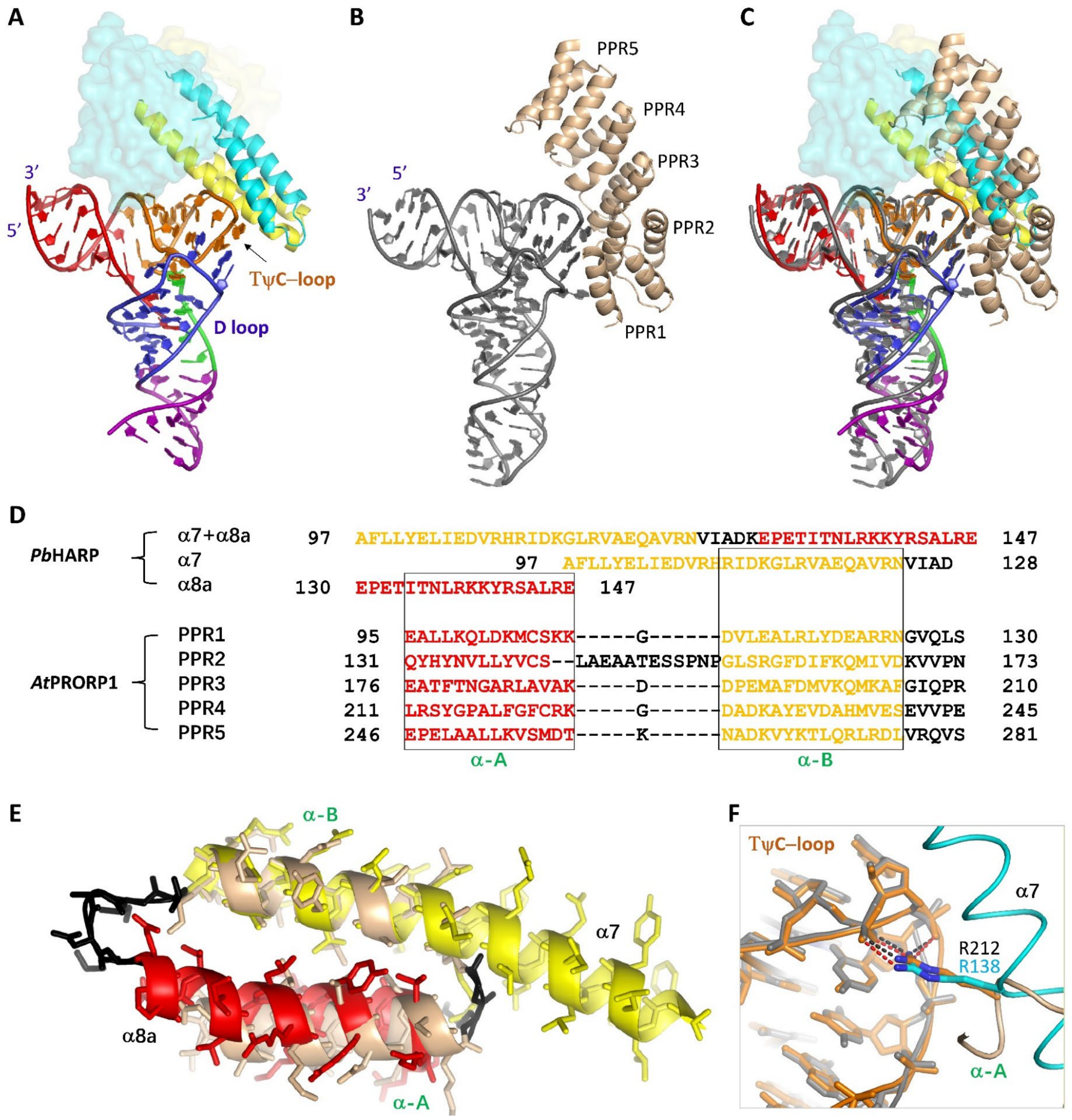

Supplementary Fig. 15: Comparison of pre-tRNA binding by different classes of PRORPs. **A**) The overall structure of *Pb*HARP/pre-tRNA^{His} complex. The metallonuclease and HB domains are shown as surface and cartoon, respectively. **B**) The overall structure of *At*PRORP1 PPR/tRNA complex (PDB_ID: 6LVR [https://www.pdbus.org/structure/6LVR]). **C**) Superposition of *Pb*HARP/pre-tRNA^{His} and *At*PRORP1 PPR/tRNA complexes. **D**) Sequence alignment of *Pb*HARP HB domain and *At*PRORP1 PPR motifs. The first (α7) and second (α8a) helices of *Pb*HARP HB domain share sequence similarity with the second (α-B) and first (α-A) helices of *At*PRORP PPR motifs, respectively. **E**) Structural comparison of *Pb*HARP HB domain and *At*PRORP1 PPR1 motif. The α7 and α8a helices of *Pb*HARP HB domain are colored in yellow and red, respectively. The α-A and α-B helices of

*At*PRORP1 PPR1 motif are colored in wheat. **F**) Conserved Arg and tRNA interactions observed in the *Pb*HARP/pre-$tRNA^{His}$ and *At*PRORP1 PPR/tRNA complexes.

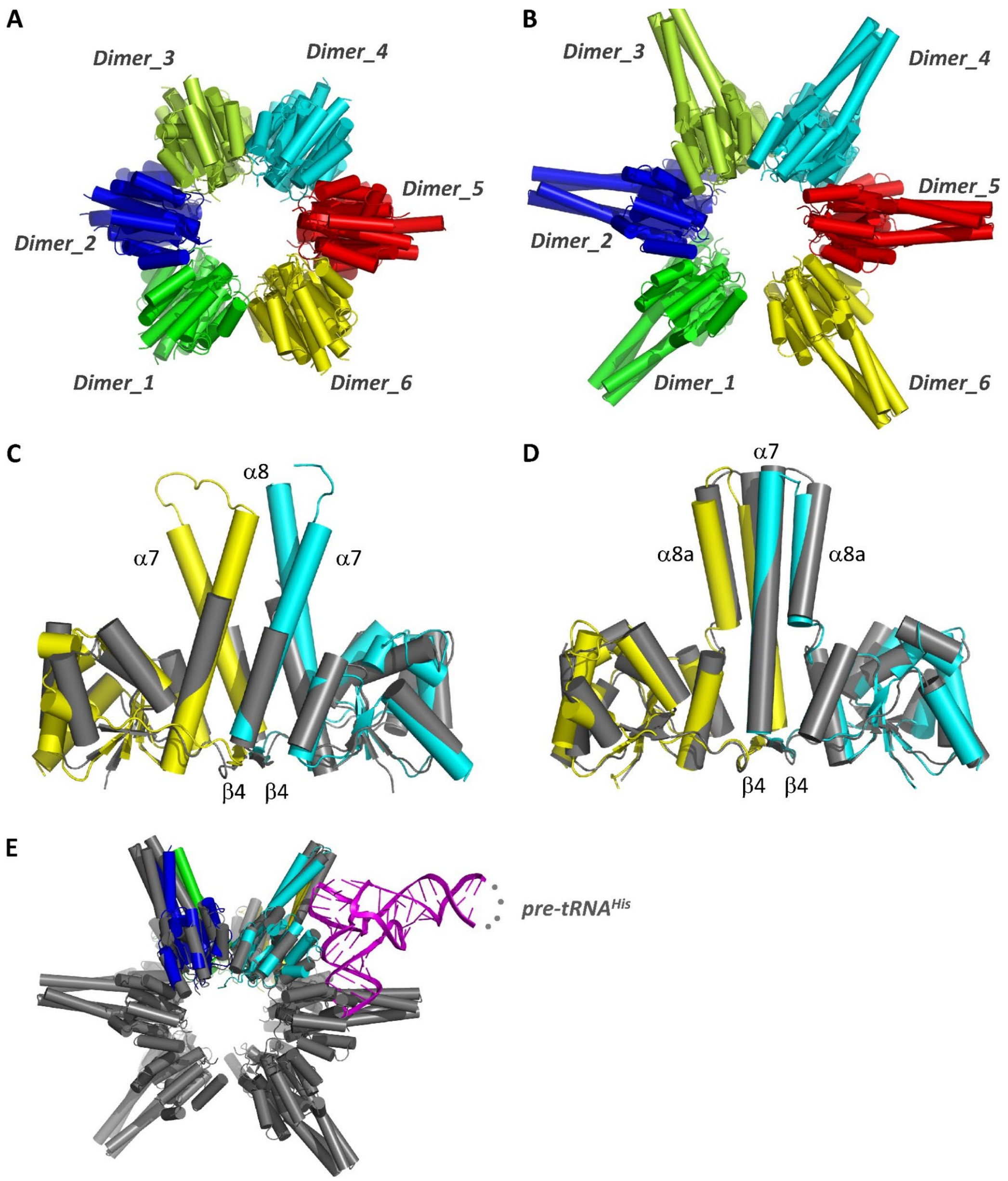

Supplementary Fig. 16: Comparison of *Pb*HARP and the reported cryo-EM structures of HARP. A) The overall structure of *Hh*HARP (PDB_ID: 7OG5 [https://www.rcsb.org/structure/7OG5]). **B**) The overall structure of Aq_880 (PDB_ID: 7F3E). **C**) Superposition of *Hh*HARP dimer and *Pb*HARP dimer in the apo-form structure. **D**) Superposition of Aq_880 dimer and *Pb*HARP dimer in the pre-tRNA^{His}-complexed structure. **E**) Superposition of *Pb*HARP/pre-tRNA^{His} complex and the Aq_880 structure. The *Hh*HARP and Aq_880 dimers are colored differently in panels **A** and **B**, whereas they are all colored in gray in panels **C-E**. The *Pb*HARP AB dimer, CD dimer, and pre-tRNA^{His} are colored in yellow and cyan, green and blue, and magenta in the *Pb*HARP/pre-tRNA^{His} complex structure, respectively.

Supplementary Table 1. Data collection and refinement statistics.

Structure	Se-*Tc*HARP	apo-*Tc*HARP	apo-*Pb*HARP	*Pb*HARP/pre-tRNA^{His} complex
PDB ID		7E8J	7E8K	7E8O
**Data collection** [a]				
Space group	$P2_12_12_1$	$P2_12_12_1$	$P3_1$	$P4_12_12$
Cell parameter:				
a (Å)	33.2	33.3	99.9	146.3
b (Å)	108.7	108.7	99.9	146.3
c (Å)	115.2	115.2	176.2	143.4
α (°)	90.0	90.0	90.0	90.0
β (°)	90.0	90.0	90.0	90.0
γ (°)	90.0	90.0	120.0	90.0
Wavelength (Å)	0.9793	0.9793	0.9793	0.9793
Resolution (Å)	50.0-2.45	50.0-2.10	30.0-2.25	30.0-2.80
Last shell (Å)	2.49-2.45	2.14-2.10	2.33-2.25	2.90-2.80
Completeness (%)	98.9(97.1)	97.0(79.9)	99.0(92.9)	99.9(99.8)
Redundancy	4.9(3.2)	7.8(2.3)	7.1(3.8)	15.1(8.0)
R_{merge} (%)	9.3(37.1)	8.7(37.4)	9.3(36.1)	11.7(59.2)
I/ σ(I)	9.2(1.8)	14.5(2.0)	17.5(2.1)	18.0(2.6)
**Refinement**				
Resolution (Å)		39.5-2.10	29.4-2.25	29.5-2.80
No. of reflections		22561	79811	33737
R_{work} (%) / R_{free} (%)		21.1/23.1	19.0/21.5	23.0/28.5
No. of atoms				
Protein		3173	6449	6147
RNA		0	0	1444
Ion		0	0	6
Water		70	373	9
R.m.s. deviations				
Bond length (Å)		0.006	0.007	0.009
Bond angle (°)		0.941	1.131	1.604
Ramachandran plot (%)				
Most favorable		98.2	98.0	97.99
Additional allowed		1.8	2.0	2.01
Outlier		0.00	0.00	0.00

a: Values in parentheses are for the last resolution shell.

Supplementary Table 2. Codon-optimized cDNA sequences of HARPs, *E. coli* RnpA and DNA sequence of *rnpB.*

**The optimized cDNA sequence of *Aquifex aeolicus* Aq_880** (from 5' to 3') [a]<br>***GGATCC***ATGGATGTGTTCGTGCTGGATACCAGCGTGTTCACCAACCCGGAAATCTACCGTACCT<br>TCGAGGAAGACCAGCGCGGTGCCATGGAAACCTTCATCCATCTGGCACTGAACAGCCGCGCCGA<br>GTTCTACATGCCGACCAGCGTGTATACCGAGATGCGCAAGATCATGGACGTGGGTGAACTGTGG<br>GCAGAGTTCGAAATGGTGGTGAAGATCCGCAGCCCGCGTCGTTTTCAGCTGACAGTTCCGGCCG<br>ATTTTTTATACGAGTTCATCGAGGAACTGCGCTACCGCATCAACAAAGGTTTACGCATTGCCGA<br>AGAACATACCCGCGAAGCAAGCGGCTGCGAAGATGTGGGCAAACTGATTGCCCGTTTACGCGAA<br>AAATATCGTGAAGCACTGCGCCAAGGTATTCTGGACAGCAAAGAAGATGTGGACGTGCTGCTGC<br>TGGCCTATGAACTGGATGGTGTGCTGGTGAGCGCCGATGAAGGTCTGCGCACTTGGGCCGATAA<br>AATTGGCATTAAACTGATTGATCCGAAGAATTTTAAAAATATTTTAGAATCTTTAGTTCGCCAC<br>CGCTTTTAA***CTCGAG***
**The optimized cDNA sequence of *Planctomycetes bacterium* HARP** (from 5' to 3') [a,b]<br>***GGATCCGGCGGCGGC***ATGCGCATGAAGAAAACTAAAAAGATTCGCGATTTAAAAGAGGAGCGCT<br>TCGTGATCGATACCAGCATTTTCACCAATACCGATGTGTACATTTTATTCGGCCGCACACCGAC<br>CACCGCTTTAAAGAACTTTCTGAAGCTGATCAGCAAGCTGAAGGGCACCAACTTCTACATGCCG<br>CCGAGCATCTACGAAGAACTGATGAATTTTATTGACAGCGATAAGATTCCGAAAGATTTACAGA<br>TTAAAATTTTTCAGAAACCGCCGAAAAAACATGAGATGGAGGTGCCGGCCTTTTTACTGTATGA<br>ACTGATCGAAGACGTGCGCCATCGCATCGATAAAGGTTTACGTGTTGCAGAACAAGCTGTGCGC<br>AATGTGATTGCCGATAAAGAGCCGGAGACCATCACCAATTTACGCAAAAAATATCGTAGCGCTT<br>TACGCGAGGGTATCATTGACAGCAAGGAAGACGTGGATTTAATTTTACTGGCCAAGGAGATGGA<br>TGGTATTCTGGTGACAGCCGATACCGGCATCATGACTTGGGCCGATAAAATGGGCATTCGCTTT<br>GTGGAAAGCCGCAATTTACGCGGCATTATCAATTCTTTAATCAAAATGTAA***CTCGAG***
**The optimized cDNA sequence of *Thermococcus celer* HARP** (from 5' to3') [a,b]<br>***GGATCCGGCGGTGGT***ATGCGCTTCGTGCTGGACACCAGCATCTTTGTGAATCCGGAAGTGCGTG<br>ATCGCTTTGGTGCCAGCCCGACCGAGGCCATGAAGACCTTTCTGGATTATGCCGGCCGTCTGTT<br>CGGCCGCGTTGAATTTTACATGCCGCCGGGCATCTATCGCGAGGTGGTGCATTTCGTGGATGAA<br>GAAGAGCTGCTGCCGGGCATTGAACTGTATATTATTAAAAAGCCGCCGAATGTGCATGACATTC<br>GCATCCCGGCCTTTGTGGTGTATGAGCTGATCGAGGATGTGCGTCGCCGCATTGATAAAGGTTT<br>ACGCGTGGCCGAAAAAGCAGTGCGCGAAAGCGTGGTGGAGACCGATAATGTGGACAAGATCATC<br>CAGAAACTGCGCCGCAACTATCGCCGTGCTTTACGTGAAGGCATTGTGGACAGCAAAGAAGATT<br>TCGAGCTGATTTTACTGGCCAAAGAACTGGATGCCACCATTGTGAGCGCCGATGTTGGCATTCT<br>GACTTGGGCCCAGAAGATGGGCATTAAATGGGTGGACGCCGCCAACTTTCGTGAGCTGCTGGAA<br>GGTTTAGTGAAAAAACTGGGCGGCAAAAATCTGTAA***CTCGAG***

The optimized cDNA sequence of *Thermocrinis minervae* HARP (from 5' to 3') [a,b]

GGATCCGGTGGTGGCATGCTGATCTTCGTGCTGGACACCAGCATCTTCACCAATCCGGATATTT
ATAGCCAGTTCGAAAAAGACCAGCTGGGCGCCATTGAGAACTTTTTATCTTTAGCCTATCACAG
CGATGCCAAGTTCTACATGCCTAGCAGCGTGTATGAGGAGATGCGCCATATGGTTGATTTAGGC
GAGCTGAGCGCCAAATTCGAGCTGACAGTGCGCATTCGCAGTCCGCGTCGCTTTAATCTGATGG
TGCCGGCCGAATTTTTATATGAGTTCATTGAAGAGATTCGCTACCGCATCAACAAGGGTCTGCG
TATTGCAGAGGAGCACACCAAAGAAGCTGGCAAACTGAGCGAAGAAGAGACCGGTAAGCTGATC
AACCGTTTACGTGAAAAATACCGCGAAGCACTGCGTACCGGCATCATCGACAGCAAAGAGGATC
TGGACGTGCTGCTGCTGGCCTATGAACTGGATGGTATTCTGGTGAGCGGTGATGAAGGCTTACG
CAAATGGGCCGATCGCGTTGGCATCAAATTAATCAACCCGAAGAATTTACGCAACATTTTAGAG
AGTCTGATTAAACATTAA***CTCGAG***

The optimized cDNA sequence of *Thermocrinis ruber* HARP (from 5' to 3') [a,b]

GGATCCGGCGGCGGCATGGAAATTTTCGTGCTGGACACCAGCGTGTTCACCAACCCGGATGTGT
ACAGCCAGTTCGAGAAAGACCAGCTGGGCGCCATTGAGAACTTTCTGTCTTTAGCCTACCACAG
CAAGGCCCAGTTTTATATGCCGCTGAGCGTTTACGAAGAATTCAATAACATGGTGTCTTTAGGC
GAGCTGAAACCGAAATTCGAACTGGTGGTTCGCATCCGTAGCCCGCGTCGCTACAATTTAATGA
TCCCGGCCGAATTTTTATATGAATTTATTGAAGAAGTGCGTTATCGCATCAATAAGGGTTTACG
TGTGGCAGAAGAACATACCCGCGAAGCCGGTCGTCTGACCGAAACCGAAACCGGCCGCGTGATT
ACCAAACTGCGCGAAAAATATCGCGAAGCTTTACGCGTGGGCATTATCGATAGCAAAGAGGATG
CCGACGTGCTGCTGCTGGCCTATGAACTGGATGCCATTTTAATTACCGGCGATGAAGGTCTGCA
TCGTTGGGCCGATCGCGTTGGCATCAAACTGATCGACCCGAAGAGCTTCCGCTATATTCTGGAA
TCTTTAGCCGGTATTCGCTAA***CTCGAG***

The optimized cDNA sequence of *E. coli* RnpA (from 5' to 3') [a,b]

GGATCCGGTGGCGGTATGGTTAAATTAGCCTTTCCGCGCGAACTGCGTCTGTTAACCCCGAGTC
AGTTTACCTTTGTGTTTCAGCAGCCGCAGCGTGCAGGAACCCCGCAGATTACCATTCTGGGTCG
CCTGAATAGCTTAGGCCATCCGCGCATTGGTCTGACCGTTGCCAAAAAAAATGTTCGCCGTGCA
CATGAACGTAATCGTATTAAACGCCTGACCCGTGAATCATTTCGCTTACGTCAGCATGAACTGC
CGGCAATGGATTTTGTGGTTGTGGCCAAAAAAGGTGTTGCCGATCTGGATAATCGTGCACTGAG
CGAAGCCCTGGAAAAATTATGGCGTCGTCATTGTCGCTTAGCACGCGGCTCTTAA***CTCGAG***

The DNA sequence of *E. coli rnpB* (from 5' to 3') [c,d,e,f]

AAGCTTTAATACGACTCACTATAGGGCTGTCTGGTCAGCTTCCTGATGAGTCCGTGAGGACGAA
ACGGTGTCGACACCGTCGAAGCTGACCAGACAGTCGCCGCTTCGTCGTCGTCCTCTTCGGGGGA
GACGGGCGGAGGGGAGGAAAGTCCGGGCTCCATAGGGCAGGGTGCCAGGTAACGCCTGGGGGGG
AAACCCACGACCAGTGCAACAGAGAGCAAACCGCCGATGGCCCGCGCAAGCGGGATCAGGTAAG
GGTGAAAGGGTGCGGTAAGAGCGCACCGCGCGGCTGGTAACAGTCCGTGGCACGGTAAACTCCA
CCCGGAGCAAGGCCAAATAGGGGTTCATAAGGTACGGCCCGTACTGAACCCGGGTAGGCTGCTT
GAGCCAGTGAGCGATTGCTGGCCTAGATGAATGACTGTCCACGACAGAACCCGGCTTATCGGTC
AGTTTCACCT***GAATTC***

[a]: ***GGATCC*** and ***CTCGAG*** at the 5'-end and 3'-end are BamHI and XhoI recognition sequence; [b]:***GGXGGXGGX***, which codes for three Gly residues, was designed to enhance the cleavage efficiency of UlP1 protease; [c]: ***AAGCTT*** and ***GAATTC*** at the 5'-end and 3'-end are HindIII and EcoRI recognition sequence; [d]:***TAATACGACTCACTATA*** is sequence of T7 promoter; [e]:***GGG*** was designed to enhance the transcriptional yield; [f]: Sequence of hammerhead ribozyme at the 5'-end of *E.coli rnpB* gene was colored in cyan.

Supplementary Table 3. Primers used for *Pb*HARP mutant construction.

Name	Sequence (from 5′ to 3′)
*Pb*HARP_F	AAAGGATCCGGCGGCGGCATG
*Pb*HARP_R	AAACTCGAGTTACATTTTGATTAAAGAATTGAT
D151A_F	CGCGAGGGTATCATTGCCAGCAAGGAAGACGTG
D151A_R	CACGTCTTCCTTGCTGGCAATGATACCCTCGCG
D155A_F	ATTGACAGCAAGGAAGCCGTGGATTTAATTTTA
D155A_R	TAAAATTAAATCCACGGCTTCCTTGCTGTCAAT
D173A_F	ATTCTGGTGACAGCCGCTACCGGCATCATGACT
D173A_R	AGTCATGATGCCGGTAGCGGCTGTCACCAGAAT
L103E/V107E_F	CTGTATGAAGAGATCGAAGACGAGCGCCATCGC
L103E/V107E_R	GCGATGGCGCTCGTCTTCGATCTCTTCATACAG
A118E/A121E_F	TTACGTGTTGAAGAACAAGAGGTGCGCAAT
A118E/A121E_R	ATTGCGCACCTCTTGTTCTTCAACACGTAA
R108A_F	CTGATCGAAGACGTGGCCCATCGCATCGATAAA
R108A_R	TTTATCGATGCGATGGGCCACGTCTTCGATCAG
R116A_F	ATCGATAAAGGTTTAGCTGTTGCAGAACAAGCT
R116A_R	AGCTTGTTCTGCAACAGCTAAACCTTTATCGAT
R123A_F	GCAGAACAAGCTGTGGCCAATGTGATTGCCGAT
R123A_R	ATCGGCAATCACATTGGCCACAGCTTGTTCTGC
T135A_F	GAGCCGGAGACCATCGCCAATTTACGCAAAAAA
T135A_R	TTTTTTGCGTAAATTGGCGATGGTCTCCGGCTC
R138A_F	ACCATCACCAATTTAGCCAAAAAATATCGTAGC
R138A_R	GCTACGATATTTTTTGGCTAAATTGGTGATGGT
R142A_F	TTACGCAAAAAATATGCTAGCGCTTTACGCGAG
R142A_R	CTCGCGTAAAGCGCTAGCATATTTTTTGCGTAA
R146A_F	TATCGTAGCGCTTTAGCCGAGGGTATCATTGAC
R146A_R	GTCAATGATACCCTCGGCTAAAGCGCTACGATA
ΔN_F	CACAGAGAACAGATTGGTGGATCCGGCGGCGGCGAGG AGCGCTTCGTGATCG
R138A/R142A_F	CACCAATTTAGCCAAAAAATATGCTAGCGCTTTAC
R138A/R142A_R	GTAAAGCGCTAGCATATTTTTTGGCTAAATTGGTG

Supplementary Table 4. Primers used for pre-tRNA and RNase P RNA template preparation.

Name	Sequence (from 5′ to 3′)
pre-tRNA^{His}_F	GCAGCGAAATTAATACGACTCACTATAGGTTGTAGTGGT GGCTATAGCTCAGTGGTAGAGCCCTGG
pre-tRNA^{His}_R	TGGGGTGGCTAATGGGATTCGAACCCACGACAACTGGAA TCACAATCCAGGGCTCTACCACTGAGC
His-7bp_F	GCAGCGAAATTAATACGACTCACTATAGGTTGTAGTTGT GGCTATAGCTCAGTGGTAGAGCCCTGG
His-6bp_F	GCAGCGAAATTAATACGACTCACTATAGGTTGTAGTTTT GGCTATAGCTCAGTGGTAGAGCCCTGG
His-9bp_F	GCAGCGAAATTAATACGACTCACTATAGGTTGTAGGGGT GGCTATAGCTCAGTGGTAGAGCCCTGG
pre-tRNA^{Cys}_F	GCAGCGAAATTAATACGACTCACTATAGGTGGCGGGTTT GGCTAGGTAACATAATGGAAATGTATCG
pre-tRNA^{Cys}_R	GGCCAAGGACGGAGTCGAACCGTCAATTCAGGATTTGCA GTCCGATACATTTCCATTATGTTACCTAG
pre-tRNA^{Phe}_F	GCAGCGAAATTAATACGACTCACTATAGGATGATCCTTC GCCGGGATAGCTCAGTTGGTAGAGCAG
pre-tRNA^{Phe}_R	GCCAAGAACCAGATTTGAACTGGTGACACGAGGATTTTC AGTCCTCTGCTCTACCAACTGAGCTATC
pre-tRNA^{Gly}_F	GCAGCGAAATTAATACGACTCACTATAGGATTTTCCCTT TCGCGGGAGTAGCTCAGTCGGTAGAGCACGAC
pre-tRNA^{Gly}_R	GACTGGAGCGGGAGACGGGACTTGAACCCGCGACCCCGA CCTTGGCAAGGTCGTGCTCTACCGACTGAGC
PUC18-HH-rnpB_F	GCAGCGAAATTAATACGACTCACTATAGGGCTGTCTGG
PUC18-HH-rnpB_R	AGGTGAAACTGACCGATAAGCCG
